# Influence of cavity margin location and preparation technique on microleakage of Class V composite restorations: An in vitro study

**DOI:** 10.4317/jced.64365

**Published:** 2026-08-28

**Authors:** Mauricio Schneider, Jefferson David Melo de Matos, Leonardo Galeazzi Farina, Letícia Krummenauer, Mário Alexandre Coelho Sinhoreti, Robert Dobrin, Daher Antonio Queiroz

**Affiliations:** 1 Department of Prosthodontics, Nova Southeastern University College of Dental Medicine, Fort Lauderdale, Florida, USA; 2 Department of Biomaterials, Dental Materials and Prosthodontics, São Paulo State University (Unesp), Institute of Science and Technology, São José dos Campos, Sao Paulo, Brazil; 3 Department of Dentistry, Lutheran University of Brazil (ULBRA), Torres, Rio Grande do Sul, Brazil; 4 Department of Restorative Dentistry, Dental Materials Division, Piracicaba Dental School (FOP UNICAMP), Piracicaba, Sao Paulo, Brazil; 5 Department of Restorative Sciences and Public Health Dentistry, Nova Southeastern University College of Dental Medicine, Fort Lauderdale, Florida, USA; 6 Department of Restorative Dentistry & Prosthodontics, UTHealth School of Dentistry, Houston, Texas, USA

**Keywords:** Microleakage, composite resins, dental cavity preparation, dentin bonding agents, ultrasonic dental instrumentation, minimally invasive dentistry.

## Abstract

**Background:**

The characteristics of the prepared dental substrate and the cavity preparation technique may influence the quality of the adhesive interface and the marginal sealing of composite restorations. Ultrasonic cavity preparation using Chemical Vapor Deposition (CVD) diamond tips has been proposed as a minimally invasive alternative to conventional rotary instrumentation. This study aimed to evaluate the influence of cavity preparation techniques and cavity margin location on the microleakage of Class V composite resin restorations.

**Materials and Methods:**

Twenty-four sound human third molars were used to prepare 48 standardized Class V cavities located at the cemento-enamel junction. Cavities were prepared using either a conventional high-speed diamond bur (KG Sorensen®) or an ultrasonic CVD diamond tip (CVDentus®). All preparations were restored with a fifth-generation etch-and-rinse adhesive system and a microhybrid composite resin. Following storage in distilled water at 37°C for seven days, specimens were immersed in 0.5% rhodamine B solution for 24 h, sectioned longitudinally, and evaluated under stereomicroscopy (40×). Microleakage was scored using a four-point ordinal scale. Data were analyzed using the Friedman nonparametric test and multiple comparisons (α=0.05).

**Results:**

The highest microleakage scores were observed in cavities with dentin margins. Mean rank values were 3.46 for the conventional bur/dentin group, 1.69 for the conventional bur/enamel group, 3.10 for the ultrasonic CVD/dentin group, and 1.75 for the ultrasonic CVD/enamel group. Groups with dentin margins showed significantly greater microleakage than enamel margins (p<0.05), whereas no significant differences were observed between conventional and ultrasonic cavity preparation (p>0.05).

**Conclusions:**

Cavity margin location significantly influenced microleakage, whereas the cavity preparation technique did not. Dentin margins exhibited significantly greater dye penetration than enamel margins regardless of the preparation method. Ultrasonic cavity preparation using CVD diamond tips demonstrated marginal sealing comparable to conventional rotary instrumentation and may be considered a viable alternative for minimally invasive restorative procedures.

## Introduction

Contemporary restorative dentistry is guided by the principles of minimally invasive dentistry, which emphasize the preservation of sound dental tissues while ensuring effective management of carious lesions and long-term restoration performance [1]. Advances in adhesive materials and restorative techniques have significantly changed the traditional concepts of cavity preparation, allowing more conservative approaches that prioritize tissue preservation and biological respect [2,3]. Consequently, the characteristics of the prepared dental substrate have become increasingly relevant because they directly influence the interaction between adhesive systems and dental tissues [2].

The success and longevity of adhesive restorations depend largely on the quality of bonding achieved with enamel and dentin [3,4]. Enamel remains the most predictable substrate for adhesion because of its high mineral content and favorable response to acid etching. In contrast, dentin presents greater bonding challenges due to its heterogeneous composition, intrinsic moisture, tubular structure, and biological relationship with the pulp tissue [4,5]. As a result, achieving durable resin-dentin bonding remains one of the major challenges in restorative dentistry despite substantial advances in adhesive technologies [3,4].

Since the introduction of acid-etching techniques by Buonocore in 1955 [6], adhesive dentistry has evolved considerably. Modern universal adhesives have simplified clinical protocols and demonstrated satisfactory clinical performance in both etch-and-rinse and self-etch approaches [4,7]. Nevertheless, the long-term stability of the adhesive interface continues to be influenced by several factors, including substrate characteristics, dentin permeability, moisture control, and the nature of the smear layer generated during cavity preparation [4,5].

The smear layer is an amorphous layer of organic and inorganic debris produced during mechanical preparation of dental tissues [5]. Its thickness, composition, and morphology vary according to the instrument and preparation technique employed. Current evidence suggests that the smear layer plays a critical role in dentin bonding, particularly when self-etch and universal adhesive systems are used [5,7]. Variations in smear layer characteristics may affect adhesive penetration, hybrid layer formation, dentin permeability, and ultimately bond durability [5].

Different cavity preparation methods produce distinct dentin surface morphologies and smear layer patterns [5,8]. Conventional high-speed rotary instruments remain the most used technique because of their efficiency and clinical familiarity. However, these instruments may generate vibration, heat, and a relatively dense smear layer that can interfere with adhesive performance [8]. Consequently, alternative preparation methods have been investigated to improve substrate quality and reduce the biological impact of operative procedures [1,8].

Ultrasonic cavity preparation has emerged as a promising minimally invasive alternative [8,9]. The development of Chemical Vapor Deposition (CVD) diamond tips represented a significant technological advance by producing a continuous diamond-coated surface with improved durability and cutting efficiency [9]. In addition to providing enhanced visibility and precision during cavity preparation, ultrasonic CVD systems have been associated with reduced noise, conservative tissue removal, and favorable surface characteristics for adhesive procedures [8,9]. Studies using scanning electron microscopy have demonstrated that ultrasonic preparation may result in cleaner dentin surfaces, reduced smear layer formation, and preservation of dentinal tubule morphology when compared with conventional rotary instrumentation [8-10].

The influence of cavity preparation techniques on marginal sealing remains clinically relevant because microleakage is strongly associated with postoperative sensitivity, marginal discoloration, secondary caries, and restoration failure [11,12]. Although contemporary adhesive systems have improved restorative outcomes, the quality of the adhesive interface remains dependent on substrate conditions established during cavity preparation [4,5]. Previous investigations have suggested that ultrasonic preparation may influence microleakage behavior; however, available evidence remains limited and sometimes contradictory, particularly regarding preparations performed with CVD diamond tips [9,10,12,13].

Although previous studies have compared ultrasonic cavity preparation with conventional rotary instrumentation using Class V microleakage models, most were conducted more than a decade ago and evaluated different ultrasonic devices, adhesive systems, and restorative materials. Furthermore, evidence regarding the effect of contemporary CVD diamond ultrasonic tips on marginal sealing remains limited. Therefore, the present study was designed to provide updated evidence using a standardized restorative protocol and current CVD technology, evaluating whether substrate modifications produced by ultrasonic preparation influence the microleakage of Class V composite restorations. Specifically, microleakage was compared between cavities prepared with conventional high-speed diamond burs (KG Sorensen®) and ultrasonic CVD diamond tips (CVDentus®) in enamel and dentin substrates. The null hypothesis was that the cavity preparation technique would not influence the degree of microleakage observed in the restored cavities.

## Materials and Methods

*Study Design and Specimen Selection*

This *in vitro* experimental study was approved by the Research Ethics Committee of the Lutheran University of Brazil (ULBRA) under protocol number 2005/079H. Twenty-four sound human third molars extracted for therapeutic reasons at the School of Dentistry of ULBRA, Torres Campus, Brazil, were included in the study.

Immediately after extraction, the teeth were cleaned of residual soft tissue and stored in 2% glutaraldehyde solution for 24 h at room temperature. Subsequently, the specimens were thoroughly rinsed and maintained in distilled water under refrigeration until use. Only teeth free of caries, restorations, cracks, or structural defects were included. No formal sample size calculation was performed because this was an exploratory *in vitro* study based on specimen availability and previous studies with similar experimental designs.

*Cavity preparation*

A total of 48 standardized Class V cavities were prepared, with two cavities per tooth, one on the buccal surface and one on the lingual surface. Each cavity measured approximately 3 mm in diameter and 2 mm in depth and was positioned at the cemento-enamel junction, involving both enamel and dentin margins.

The allocation of cavities to the preparation techniques was performed using a computer-generated random sequence.

Conventional rotary preparation: Performed using a #1014 diamond bur (KG Sorensen®, Cotia, SP, Brazil) mounted on a high-speed handpiece (Kavo®, Germany) under continuous water cooling.

Ultrasonic preparation: Performed using a CVDentus® diamond tip (CVDentus®, São José dos Campos, SP, Brazil) coupled to an ultrasonic device (Gnatus®, Ribeirão Preto, SP, Brazil), according to the manufacturer's recommendations.

*Experimental Design*

All cavity preparations were etched with 35% phosphoric acid gel (Magic Acid®, Vigodent, Rio de Janeiro, Brazil) following the manufacturer's instructions. The cavities were then rinsed and gently air-dried.

A fifth-generation etch-and-rinse adhesive system (Magic Bond®, Vigodent, Rio de Janeiro, Brazil) was applied according to the manufacturer's instructions and light-cured.

The cavities were restored using a microhybrid composite resin (Concept®, Vigodent, shade A3). Resin insertion was performed using the incremental technique in three successive layers. Each increment was individually light-cured using a LED curing unit (3M ESPE, St. Paul, MN, USA) according to the manufacturer's recommended exposure time.

After polymerization, restorations were finished and polished using Sof-Lex™ abrasive discs (3M ESPE, St. Paul, MN, USA):

*Microleakage Assessment*

The root apices of all specimens were sealed with two layers of epoxy resin (Araldite®, Brascola Ltda., São Bernardo do Campo, SP, Brazil). Subsequently, the entire tooth surface was coated with two layers of nail varnish, leaving a 1- mm window around the restoration margins exposed.

The specimens were stored in distilled water at 37°C for seven days. After this period, the teeth were immersed in 0.5% rhodamine B solution for 24 h at 37°C to allow dye penetration.

Following dye exposure, the teeth were rinsed under running water and sectioned longitudinally through the center of each restoration using a diamond disc (22 mm diameter × 0.25 mm thickness; Superflex®, Dental Prudent, USA) under water cooling. The sectioning procedure produced two evaluable sections per restoration.

*Experimental Groups*

The specimens were classified according to the preparation technique and cavity margin location:

BD Group: Diamond bur preparation - dentin margin.

BE Group: Diamond bur preparation - enamel margin.

UD Group: Ultrasonic CVD preparation - dentin margin.

UE Group: Ultrasonic CVD preparation - enamel margin.

*Microscopic Evaluation*

Microleakage was assessed by a calibrated examiner blinded to the experimental groups. Intra-examiner agreement was evaluated using Cohen's kappa coefficient = 0.87. The sections were examined under a stereomicroscope at 40× magnification.

Dye penetration was scored according to the following criteria:

Score 0: No dye penetration.

Score 1: Dye penetration extending up to half of the cavity wall.

Score 2: Dye penetration extending along the entire cavity wall.

Score 3: Dye penetration extending along the cavity wall and reaching the axial wall.

Representative images of cavity preparations performed with conventional diamond burs and ultrasonic CVD tips are shown in Figures 1 and 2, respectively.

*Statistical analysis*

Microleakage scores were analyzed using the Friedman nonparametric test. Statistical analyses were performed at a significance level of 5% (α = 0.05). Statistical analyses were performed using SPSS version 22.0 (IBM Corp., Armonk, NY, USA).

## Results

The distribution of microleakage scores according to the cavity preparation technique and substrate is presented in Table 1 and Figure 3.

No specimens in the BD group exhibited scores 0, 1, or 2. All specimens in this group showed dye penetration reaching the axial wall (score 3). In the UD group, most specimens also presented severe microleakage, with 19 of the 24 specimens classified as score 3. In contrast, the BE and UE groups demonstrated lower microleakage scores, with a higher frequency of specimens exhibiting no dye penetration (score 0) or dye penetration restricted to the cavity wall (scores 1 and 2).

The mean rank values obtained for each experimental group were 3.46 for BD, 1.69 for BE, 3.10 for UD, and 1.75 for UE.

Statistical analysis using the Friedman nonparametric test followed by multiple comparisons revealed significant differences among the experimental groups (*p=*0.003). The BD and UD groups exhibited significantly higher microleakage scores than the BE and UE groups. No significant differences were observed between BD and UD, nor between BE and UE (*p>*0.05).

These findings indicate that cavities with dentin margins showed significantly greater microleakage than those with enamel margins, regardless of the preparation technique employed (Figure 3).

## Discussion

Microleakage remains one of the main challenges in adhesive restorative dentistry because it is associated with postoperative sensitivity, marginal discoloration, recurrent caries, and reduced restoration longevity. Although substantial improvements have been achieved in adhesive systems and restorative materials over the last decades, complete elimination of marginal leakage remains difficult, particularly when restoration margins are in dentin or cementum rather than enamel [14,15].

In the present study, the most important finding was that the location of the cavity margin had a greater influence on microleakage than the cavity preparation technique itself. Significantly higher microleakage scores were observed at dentin margins compared with enamel margins, regardless of whether the cavities were prepared using conventional diamond burs or ultrasonic CVD tips. These findings are consistent with previous studies reporting superior marginal sealing in enamel due to its highly mineralized and homogeneous structure, which favors predictable resin infiltration and micromechanical retention following acid etching [16-18].

The greater microleakage observed in dentin margins can be explained by the intrinsic characteristics of dentin, including its organic composition, tubular structure, permeability, and moisture content. Unlike enamel, dentin is a biologically dynamic substrate that presents additional challenges for adhesive procedures. Consequently, the formation and long-term stability of the hybrid layer may be compromised, increasing the risk of marginal leakage and degradation of the adhesive interface over time [19,20].

Another relevant finding was the absence of statistically significant differences between the cavity preparation techniques. The BD and UD groups showed similar microleakage behavior, as did the BE and UE groups. This suggests that the use of ultrasonic CVD tips does not negatively affect the marginal sealing ability of composite restorations when compared with conventional rotary instrumentation. A possible explanation is that the phosphoric acid etching procedure, thereby reducing potential differences between the substrates prior to adhesive application employed in this study may have minimized the morphological differences originally created by the preparation techniques through the removal of the smear layer and exposure of the underlying dental substrate before adhesive application [19,21].

Previous investigations have demonstrated that ultrasonic preparation may produce cleaner dentin surfaces, reduced smear layer formation, and greater preservation of dentinal tubule morphology compared with conventional burs [8,9]. However, despite these morphological differences, the present findings indicate that such surface modifications were not sufficient to produce significant differences in microleakage after adhesive restoration. Similar observations have been reported in studies evaluating alternative cavity preparation methods, where differences in surface morphology did not necessarily translate into differences in marginal sealing performance [19-22].

The methodology adopted in this study did not include thermocycling. Although thermal cycling is frequently used in laboratory studies to simulate intraoral temperature variations, its influence on microleakage outcomes remains controversial. Several investigations have reported limited or no significant effect of thermocycling on marginal leakage patterns, particularly when contemporary adhesive systems are employed [22,23]. Therefore, the storage of specimens in distilled water at 37°C was considered adequate for standardization of the experimental conditions.

From a clinical perspective, the findings suggest that ultrasonic cavity preparation using CVD diamond tips may be considered a viable alternative to conventional rotary instrumentation. In addition to producing comparable microleakage results, ultrasonic systems have been associated with improved operative control, reduced noise, lower vibration, and greater patient comfort, which may be advantageous during minimally invasive restorative procedures [21,24,25].

A strength of the present study was the direct comparison between conventional rotary instrumentation and ultrasonic CVD cavity preparation under standardized restorative conditions, allowing the isolated evaluation of their influence on microleakage. This design enabled a direct assessment of the effect of the preparation technique while minimizing the influence of confounding variables related to restorative procedures and adhesive protocols.

The present findings should be interpreted as confirmatory rather than disruptive. Previous studies by Setien *et al*. [24] and Vieira *et al*. [25] similarly reported that cavity preparation method exerted limited influence on microleakage when compared with the effect of the bonding substrate. Nevertheless, technological developments in ultrasonic instrumentation and adhesive dentistry justify the reassessment of this relationship using contemporary restorative materials and CVD diamond technology. The present study contributes updated evidence demonstrating that the previously observed trend remains valid under current restorative conditions.

The limitations of this study should be acknowledged. First, the *in vitro* design cannot completely reproduce the biological, thermal, and mechanical challenges present in the oral environment. No thermocycling procedure was performed, which may limit extrapolation of the findings to long-term clinical performance. However, the study was designed to isolate the influence of cavity preparation technique and margin location under standardized laboratory conditions before introducing additional aging variables. Second, no a priori sample size calculation was performed because the study had an exploratory design based on specimen availability and methodological similarity to previous investigations. Consequently, the results should be interpreted with caution regarding statistical power and external validity. Third, only one adhesive system and one composite resin were evaluated, limiting the generalizability of the findings to other contemporary adhesive strategies such as self-etch and universal systems. Future studies incorporating thermocycling, mechanical loading, larger sample sizes, and different adhesive materials are recommended to further investigate the clinical relevance of these findings.

## Conclusions

Within the limitations of this *in vitro* study, it can be concluded that: No significant differences in microleakage were observed between cavities prepared with conventional diamond burs and those prepared with ultrasonic CVD tips. Cavities with dentin margins exhibited significantly higher microleakage scores than cavities with enamel margins, regardless of the preparation technique employed. The cavity preparation technique did not significantly influence the marginal sealing ability of the adhesive restorations evaluated. Ultrasonic cavity preparation using CVD diamond tips may be considered a viable alternative to conventional rotary instrumentation with respect to microleakage performance. 

## Figures and Tables

**Table 1:** Distribution of microleakage scores and mean rank values according to cavity preparation technique and substrate. BD: Cavity preparation with conventional diamond bur in dentin; BE: Cavity preparation with conventional diamond bur in enamel; UD: Cavity preparation with ultrasonic CVD tip in dentin; UE: Cavity preparation with ultrasonic CVD tip in enamel. Different uppercase letters indicate statistically significant differences according to the Friedman test followed by multiple comparisons (p=0.003).

**Infiltration levels**	**Group**			
	BD	BE	UD	UE
No dye penetration	0	3	2	3
Dye penetration up to half of the cavity wall	0	13	2	12
Dye penetration along the entire cavity wall	0	5	1	5
Dye penetration reaching the axial wall	24	3	19	4
Total	24	24	24	24
Mean rank	3.46^ A^	1.69^ B^	3.10^ A^	1.75^ B^

**Figure 1:**
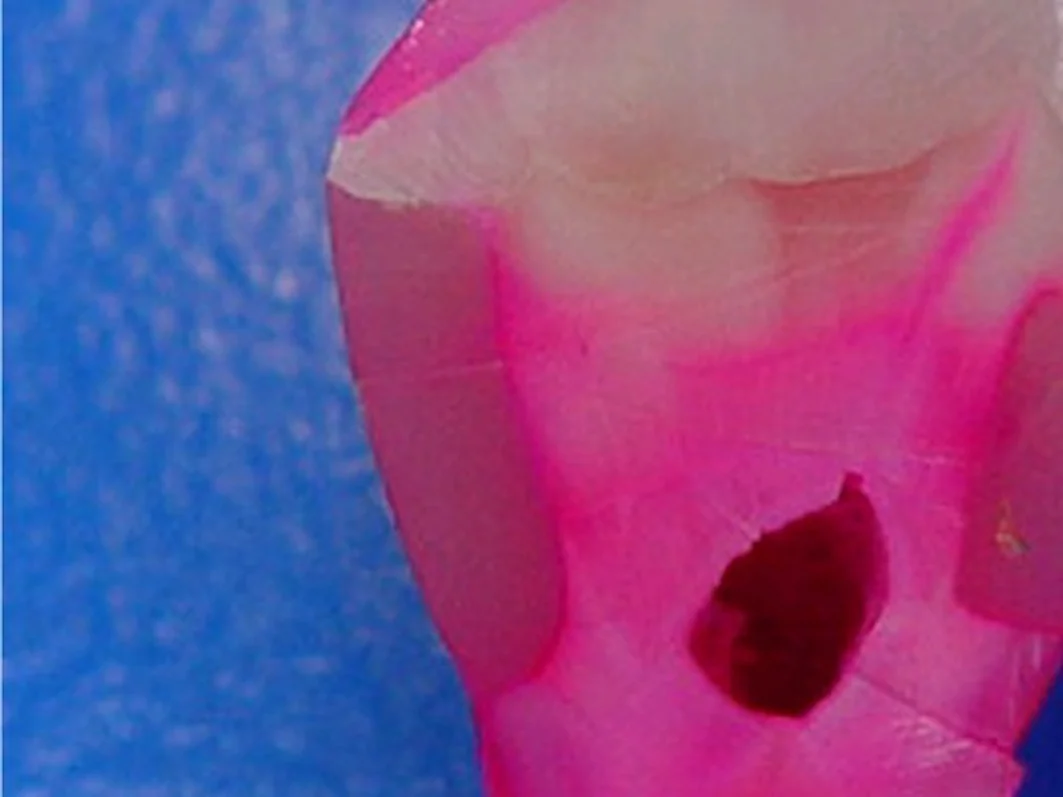
Representative image of Class V cavity preparation performed using a conventional high-speed diamond bur (#1014 diamond bur, KG Sorensen®).

**Figure 2:**
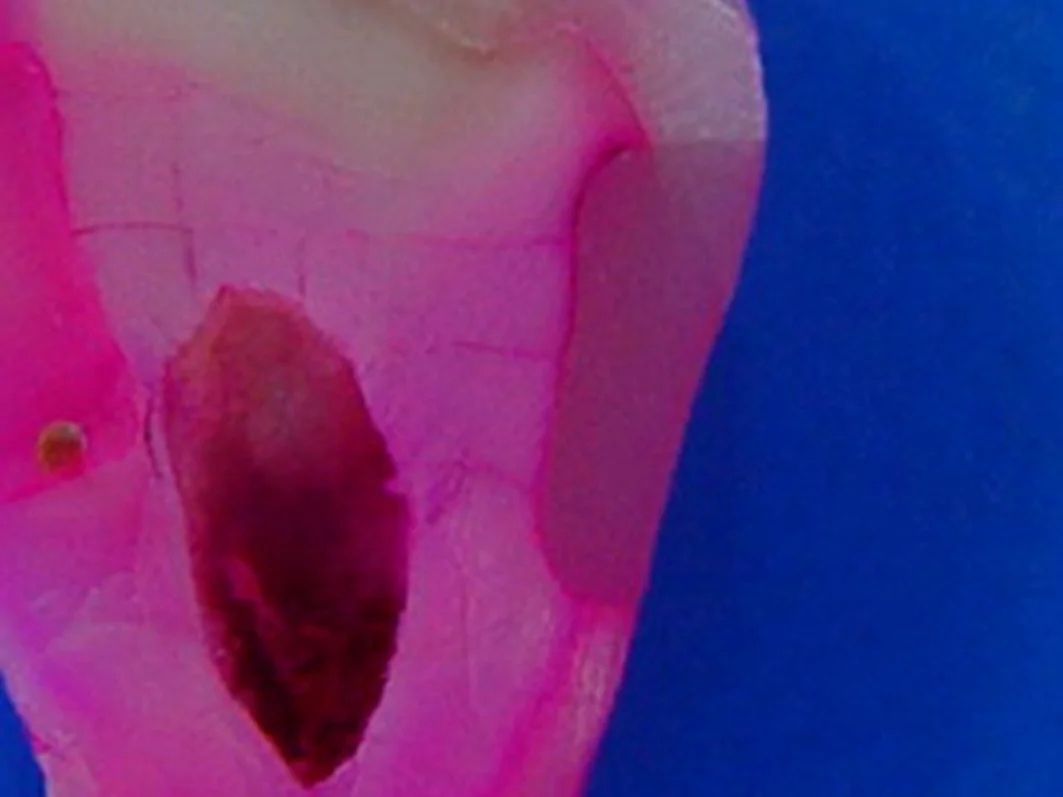
Representative image of Class V cavity preparation performed using an ultrasonic device equipped with a CVD diamond tip (CVDentus®).

**Figure 3:**
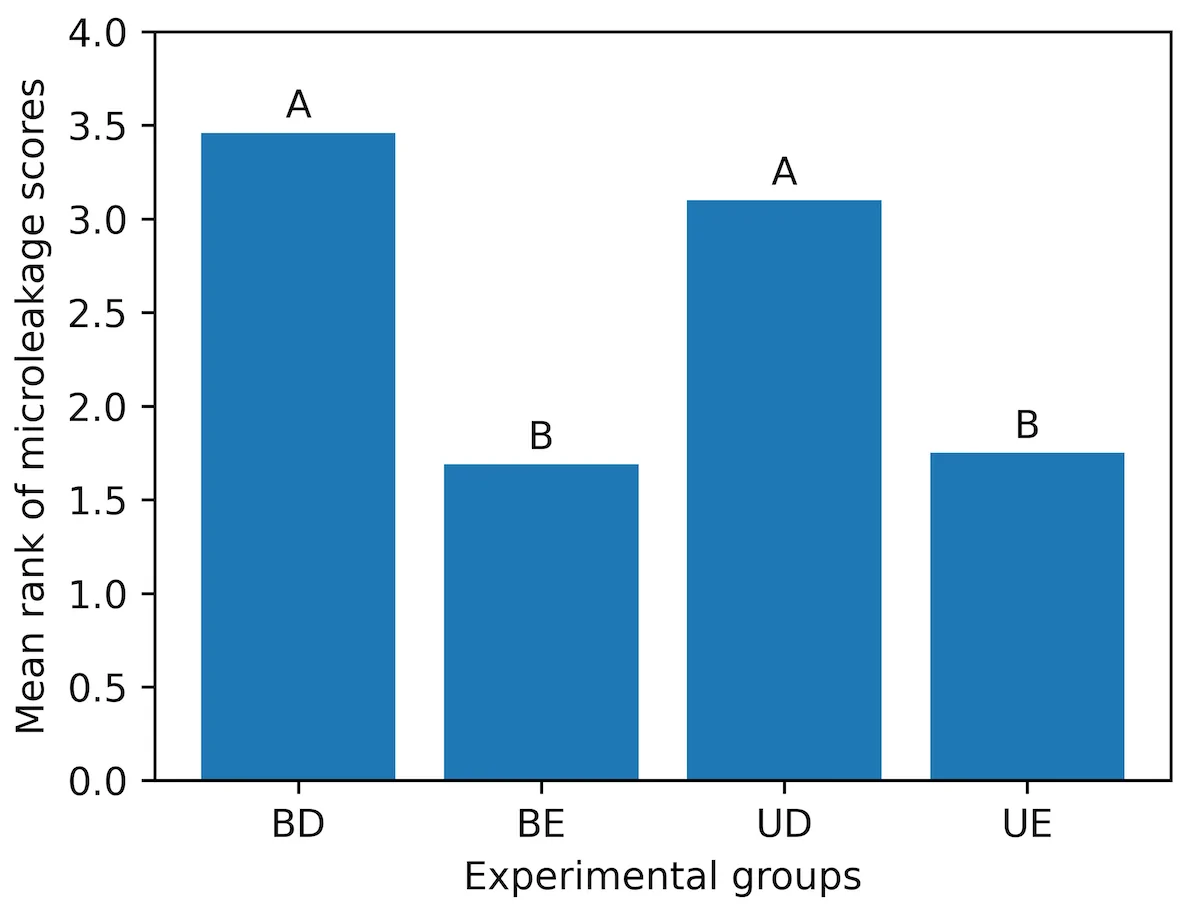
Mean rank values of microleakage scores according to the experimental groups. BD: Cavity preparation with conventional diamond bur in dentin; BE: Cavity preparation with conventional diamond bur in enamel; UD: Cavity preparation with ultrasonic CVD tip in dentin; UE: Cavity preparation with ultrasonic CVD tip in enamel. Different uppercase letters indicate statistically significant differences according to the Friedman test followed by multiple comparisons (p<0.05).

## Data Availability

The datasets generated and analyzed during the current study are available from the corresponding author upon reasonable request.
